# Prevalence and causes of low vision and blindness in a Chinese population with type 2 diabetes: the Dongguan Eye Study

**DOI:** 10.1038/s41598-017-11365-z

**Published:** 2017-09-11

**Authors:** Ying Cui, Liang Zhang, Min Zhang, Xiaohong Yang, Lixin Zhang, Jian Kuang, Guanrong Zhang, Qingyang Liu, Haike Guo, Qianli Meng

**Affiliations:** 1 Guangdong Eye Institute, Department of Ophthalmology, Guangdong General Hospital, Guangdong Academy of Medical Sciences, Guangzhou, Guangdong China; 2 Department of Ophthalmology, Dongguan People’s Hospital, Dongguan, Guangdong China; 3 Department of Ophthalmology, Hengli Hospital, Dongguan, Guangdong China; 4 Department of Endocrinology, Guangdong General Hospital, Guangdong Academy of Medical Sciences, Guangzhou, Guangdong China; 5 Health Management Center, Guangdong General Hospital, Guangdong Academy of Medical Sciences, Guangzhou, Guangdong China; 6 Xiamen Eye Centre of Xiamen University, Xiamen, China

## Abstract

To assess the prevalence and causes of low vision and blindness in type 2 diabetes patients, a population-based cross-sectional study including 8952 rural-dwelling residents aged 40 years or older from Hengli Town in Southern China was conducted. Participants underwent standard interviews, physical measurements, laboratory tests, and comprehensive eye examinations. Low vision and blindness were defined based on WHO criteria. Visual acuity data were available for 1348 (89.9%) of the 1500 subjects with type 2 diabetes. Age-standardized prevalence of bilateral low vision and blindness assessed in the better-seeing eye was 2.9% (95% confidence interval [CI]: 2.0–3.8) and 0.7% (95% CI: 0.2–1.1) based on best-corrected visual acuity (BCVA). Cataracts were the primary cause of low vision and blindness. Visual impairment was associated with age (odds ratio [OR]: 3.73, 95% CI: 2.39–5.83), education level (OR: 3.21, 95% CI: 1.63–6.29), duration of diabetes (OR: 1.14, 95% CI: 1.04–1.25) and body mass index (OR: 0.86, 95% CI: 0.77–0.95). Our data suggest that approximately 70% of visual impairment in this diabetic population could be eliminated with appropriate cataract surgery or spectacle correction. Greater consideration should be given to older type 2 diabetes patients with a level of lower education.

## Introduction

Diabetes mellitus is often associated with progressive loss of vision and is one of the fastest-growing health problems in the world^1, 2^. The loss of vision is more likely to occur in people with type 2 diabetes than in older adults without type 2 diabetes^3, 4^. This has been explained based on the presence of proliferative diabetic retinopathy and clinically significant macular edema. Moreover, a higher frequency of cataracts, glaucoma, or corneal diseases has also been observed in this population^5–8^. Visual impairment not only aggravates the reduced quality of life of diabetic patients but also increases the economic burden on society. The increased prevalence of visual impairment in association with diabetes has become a major public health problem that requires significant attention.

It should be noted that as the most populous developing country, China is entering an accelerated period of urbanization, and the country’s population distribution and economic structure are undergoing tremendous changes, with concomitant increases in the prevalence of diabetes mellitus. A national survey conducted from 2007–2008 showed that the prevalence of diabetes and pre-diabetes in individuals aged 20 years or older was 9.7% and 15.2%, respectively, accounting for 92.4 million adults with diabetes and 148.2 million adults with prediabetes^9^. The rapid transition in urbanization and the large increase in the prevalence of diabetes mellitus inevitably affects the pattern of visual impairment in Chinese populations, especially in diabetic patients. Currently, little is known about how low vision and blindness are associated with type 2 diabetes in China. Thus, we conducted this study to determine the prevalence and causes of low vision and blindness in a population with type 2 diabetes screened from the Dongguan Eye Study (DES).

## Results

### Baseline characteristics of participants with type 2 diabetes

All eligible participants (8952) were self-identified Han Chinese, and 59.9% were female. The average age was 54.0 years (range: 46.0–62.0); 87.2% of individuals ranged in age from 40 to 69 years old, 48.4% were farmers, and 77.2% had elementary or junior middle school levels of education. The average body mass index (BMI) and waist-hip ratio were 24.6 ± 3.9 kg/m^2^ and 0.88 ± 0.23, respectively. Fifteen hundred subjects were diagnosed as having type 2 diabetes, for a prevalence of 16.8%. In this diabetic population, there were no statistically significant differences between the participants (1348/1500) and the non-participants (152/1500) in terms of demographic, physical, and blood glucose characteristics (P > 0.05) (Table 1).

**Table 1:** Demographic and physical characteristics of participants and nonparticipants in the type 2 diabetes population of the Dongguan Eye Study.

Variables	Non-participants (n = 152) No. of persons (%) or Mean ± SD	Participants (n = 1348) No. of persons (%) or Mean ± SD	*P*
Gender			0.276
Male	69 (45.4)	550 (40.8)	
Female	83 (54.6)	798 (59.2)	
Age (yrs)			
Mean ± SD	58.7 ± 13.6	59.6 ± 11.0	0.458
40–49	44 (29.0)	296 (22.0)	0.183
50–59	40 (26.3)	390 (28.9)	
60–69	39 (25.7)	419 (31.1)	
70–79	18 (11.8)	177 (13.1)	
≥80	11 (7.2)	66 (4.9)	
Education			0.827
High school and above	21 (13.8)	151 (11.2)	
Junior middle school	48 (31.6)	422 (31.3)	
Primary school	58 (38.2)	657 (48.7)	
No education	25 (16.5)	118 (8.8)	
Occupation			0.348
Farmers	86 (56.6)	648 (48.1)	
Workers	8 (5.3)	102 (7.6)	
Technicians	1 (0.7)	20 (1.5)	
Civil servants	2 (1.3)	26 (1.9)	
Self-employed	8 (5.3)	47 (3.5)	
Retirees	29 (19.1)	314 (23.3)	
Others	18 (11.8)	191 (14.2)	
BMI (kg/m^2^)	26.1 ± 4.2	26.2 ± 3.8	0.647
Waist-hip-ratio	0.9 ± 0.1	0.9 ± 0.1	0.211
Mean SBP (mmHg)	143.6 ± 24.6	141.6 ± 20.1	0.341
Mean DBP (mmHg)	79.8 ± 12.2	78.4 ± 11.0	0.199
FBG (mmol/l)	7.6 ± 2.9	7.6 ± 2.9	0.861
HbA1c (%)	7.0 ± 1.6	7.1 ± 1.7	0.918

SD: Standard deviation; BMI: body mass index; SBP: systolic blood pressure; DBP: diastolic blood pressure; FBG: fasting blood glucose; HbA1C: glycosylated haemoglobin. BMI = weight (kg)/height (m^2^). Waist-hip-ratio = waist circumference (cm)/hip circumference (cm).

For the 1348 participants with diabetes, the average age was 59.6 ± 11.0 years (range: 40–96; 95% confidence interval [CI]: 59.0–60.2), and 59.2% were women. Approximately 57.5% of the participants had a primary school level of education or were illiterate, and 48.1% were farmers (Table 1). There were 950 individuals with newly diagnosed type 2 diabetes. Compared with the non-diabetic population in the DES, the type 2 diabetes patients not only had higher blood glucose levels but also were older, heavier, had higher BMI scores, waist-hip ratios, and blood pressure, and had abnormal blood lipid levels.

### Prevalence of low vision and blindness

According to best-corrected visual acuity (BCVA) results, the age-standardized prevalence of bilateral low vision was 2.9% (95% CI: 2.0–3.8), and the prevalence of bilateral blindness was 0.7% (95% CI: 0.2–1.1). The age-standardized prevalence of bilateral low vision and blindness based on presenting visual acuity (PVA) increased to 8.2% (95% CI: 6.8–9.5) and 0.8% (95% CI: 0.3–1.3), respectively. Table 2 shows the crude prevalence of bilateral low vision and blindness by gender and age in this diabetic population. Among participants aged 60 years or older, the prevalence of bilateral low vision was 5.6% (37/662, 95% CI: 3.8–7.3) and that of blindness was 1.2% (8/662, 95% CI: 0.4–2.0) based on BCVA, while low vision was present in 14.2% (94/662, 95% CI: 11.5–16.9) and blindness in 1.5% (10/662, 95% CI: 0.6–2.4) based on PVA.

**Table 2:** Crude prevalence of bilateral low vision and blindness based on best-corrected visual acuity (BCVA) or presenting visual acuity (PVA) by gender and age in type 2 diabetes patients in the Dongguan Eye Study.

Group	Age	Total No. of persons	Based on BCVA				Based on PVA			
			Bilateral low vision (20/400 ≤BCVA < 20/63)		Bilateral blindness (BCVA < 20/400)		Bilateral low vision (20/400 ≤ PVA < 20/63)		Bilateral blindness (PVA < 20/400)	
			No. of persons	% (95% CI)	No. of persons	% (95% CI)	No. of persons	% (95% CI)	No. of persons	% (95% CI)
Male										
	40–49	156	0	0	0	0	1	0.6 (0–1.9)	0	0
	50–59	168	0	0	1	0.6 (0–1.8)	4	2.4 (0.1–4.7)	1	0.6 (0–1.8)
	60–69	158	2	1.3 (0–3.0)	1	0.6 (0–1.9)	5	3.2 (0.4–5.9)	1	0.6 (0–1.9)
	70–79	46	2	4.3 (0–10.3)	1	2.2 (0–6.4)	11	23.9 (11.4–36.4)	1	2.2 (0–6.4)
	≥80	22	5	22.7 (4.8–40.7)	2	9.1 (0–21.4)	6	27.3 (8.2–46.4)	2	9.1 (0–21.4)
	total	550	9	1.6 (0.6–2.7)	5	0.9 (0.1–1.7)	27	4.9 (3.1–6.7)	5	0.9 (0.1–1.7)
Female										
	40–49	140	0	0	0	0	4	2.9 (0.1–5.6)	0	0
	50–59	222	1	0.5 (0–1.3)	0	0	6	2.7 (0.6–4.8)	0	0
	60–69	261	3	1.1 (0–2.4)	0	0	17	6.5 (3.5–9.5)	1	0.4 (0–1.1)
	70–79	131	17	13.0 (7.2–18.8)	0	0	40	30.5 (22.6–38.5)	0	0
	≥80	40	8	18.2 (6.6–29.7)	4	9.1 (0.5–17.7)	15	34.1 (19.9–48.3)	5	11.4 (1.9–20.9)
	total	798	29	3.6 (2.3–4.9)	4	0.5 (0–1.0)	82	10.3 (8.2–12.4)	6	0.8 (0.2–1.4)
Male and female										
	40–49	296	0	0	0	0	5	1.7 (0.2–3.2)	0	0
	50–59	390	1	0.3 (0–0.8)	1	0.3 (0–0.8)	10	2.6 (1.0–4.1)	1	0.3 (0–0.8)
	60–69	419	5	1.2 (0.2–2.2)	1	0.2 (0–0.7)	22	5.3 (3.1–7.4)	2	0.5 (0–1.1)
	70–79	177	19	10.7 (6.2–15.3)	1	0.6 (0–1.7)	51	28.8 (22.1–35.5)	1	0.6 (0–1.7)
	≥80	66	13	19.7 (10.0–29.4)	6	9.1 (2.1–16.1)	21	31.8 (20.5–43.2)	7	10.6 (3.1–18.1)
	total	1348	38	2.8 (1.9–3.7)	9	0.7 (0.2–1.1)	109	8.1 (6.6–9.5)	11	0.8 (0.3–1.3)

Based on BCVA in the worse-seeing eye, the age-standardized prevalence of unilateral low vision and blindness was 4.7% (95% CI: 3.6–5.8) and 2.8% (95% CI: 1.9–3.6), respectively, which increased to 8.5% (95% CI: 7.1–10.0) and 3.3% (95% CI: 2.4–4.2) based on PVA. A higher prevalence of vision impairment was also observed in individuals aged 60 years and older (13.4% [89/662, 95% CI: 10.8–16.0] based on BCVA, 18.6% [123/662, 95% CI: 15.6–21.5] based on PVA) (Table 3).

**Table 3:** Crude prevalence of unilateral low vision and blindness based on best-corrected visual acuity (BCVA) or presenting visual acuity (PVA) by gender and age in type 2 diabetes patients in the Dongguan Eye Study.

Group	Age	Total No. of persons	Based on BCVA				Based on PVA			
			Unilateral low vision (20/400 ≤ BCVA < 20/63)		Unilateral blindness (BCVA < 20/400)		Unilateral low vision (20/400 ≤ PVA < 20/63)		Unilateral blindness (PVA < 20/400)	
			No. of persons	% (95% CI)	No. of persons	% (95% CI)	No. of persons	% (95% CI)	No. of persons	% (95% CI)
Male										
	40–49	156	1	0.6 (0–1.9)	1	0.6 (0–1.9)	5	3.2 (0.4–6.0)	1	0.6 (0–1.9)
	50–59	168	4	2.4 (0.1–4.7)	1	0.6 (0–1.8)	6	3.6 (0.8–6.4)	3	1.8 (0–3.8)
	60–69	158	3	1.9 (0–4.0)	5	3.2 (0.4–5.9)	11	7.0 (3.0–11.0)	5	3.2 (0.4–5.9)
	70–79	46	8	17.4 (6.3–28.5)	2	4.3 (0–10.3)	8	17.4 (6.3–28.5)	2	4.3 (0–10.3)
	≥80	22	3	13.6 (0–28.3)	5	22.7 (4.8–40.7)	4	18.2 (1.6–34.7)	4	18.2 (1.6–34.7)
	total	550	19	3.5 (1.9–5.0)	14	2.5 (1.2–3.9)	34	6.2 (4.2–8.2)	15	2.7 (1.4–4.1)
Female										
	40–49	140	1	0.7 (0–2.1)	0	0	7	5.0 (1.4–8.6)	0	0
	50–59	222	7	3.2 (0.8–5.5)	0	0	17	7.7 (4.1–11.2)	2	0.9 (0–2.1)
	60–69	261	16	6.1 (3.2–9.1)	10	3.8 (1.5–6.2)	29	11.1 (7.3–14.9)	10	3.8 (1.5–6.2)
	70–79	131	16	12.2 (6.6–17.9)	9	6.9 (2.5–11.2)	20	15.3 (9.1–21.5)	13	9.9 (4.8–15.1)
	≥80	40	6	13.6 (3.4–23.9)	6	13.6 (3.4–23.9)	11	25.0 (12.0–38.0)	6	13.6 (3.4–23.9)
Male and female										
	40–49	296	2	0.7 (0–1.6)	1	0.3 (0–1.0)	12	4.1 (1.8–6.3)	1	0.3 (0–1.0)
	50–59	390	11	2.8 (1.2–4.5)	1	0.3 (0–0.8)	23	5.9 (3.6–8.2)	5	1.3 (0.2–2.4)
	60–69	419	19	4.5 (2.5–6.5)	15	3.6 (1.8–5.4)	40	9.5 (6.7–12.4)	15	3.6 (1.8–5.4)
	70–79	177	24	13.6 (8.5–18.6)	11	6.2 (2.6–9.8)	28	15.8 (10.4–21.2)	15	8.5 (4.4–12.6)
	≥80	66	9	13.6 (5.3–22.0)	11	16.7 (7.6–25.7)	15	22.7 (12.5–32.9)	10	15.2 (6.4–23.9)
	total	1348	65	4.8 (3.7–6.0)	39	2.9 (2.0–3.8)	118	8.8 (7.2–10.3)	46	3.4 (2.4–4.4)

### Causes of low vision and blindness

As shown in Table 4, the most common cause of bilateral low vision and blindness based on BCVA was cataracts (cataracts were grouped with posterior capsular opacification in pseudophakic and aphakic eyes), which accounted for 76.3% (29/38) and 55.6% (5/9) of cases respectively. A retinal disease other than diabetic retinopathy and age-related maculopathy (ARM), responsible for 8.5% (4/47) of cases, was the second most common cause of visual impairment. Diabetic retinopathy (including 2 cases of low vision and 1 of blindness) and pterygium (including 3 cases of low vision) were tied as the third most common cause (6.4%), followed by myopic maculopathy, corneal opacity and ARM (2.1%).

**Table 4:** Causes of bilateral low vision and blindness based on best-corrected visual acuity (BCVA) or presenting visual acuity (PVA) in type 2 diabetes patients in the Dongguan Eye Study (per person).

Causes	Bilateral low vision and blindness based on BCVA			Bilateral low vision and blindness based on PVA		
	Bilateral low vision (BCVA < 20/63 and ≥20/400) No. at risk (%)	Bilateral blindness (BCVA < 20/400) No. at risk (%)	Bilateral visual impairment (BCVA < 20/63) No. at risk (%)	Bilateral low vision (PVA < 20/63 and ≥20/400) No. at risk (%)	Bilateral blindness (PVA < 20/400) No. at risk (%)	Bilateral visual impairment (PVA < 20/63) No. at risk (%)
Cataract	29 (76.3)	5 (55.6)	34 (72.3)	66 (60.6)	5 (45.5)	71 (59.2)
Other retinal disease	3 (7.9)	1 (11.1)	4 (8.5)	4 (3.7)	1 (9.1)	5 (4.2)
Diabetic retinopathy	2 (5.3)	1 (11.1)	3 (6.4)	2 (1.8)	1 (9.1)	3 (2.5)
Pterygium	3 (7.9)	0	3 (6.4)	4 (3.7)	0	4 (3.3)
Myopic maculopathy	1 (2.6)	0	1 (2.1)	1 (0.9)	0	1 (0.8)
Corneal opacity	0	1 (11.1)	1 (2.1)	0	1 (9.1)	1 (0.8)
ARM	0	1 (11.1)	1 (2.1)	1 (0.9)	1 (9.1)	2 (1.7)
Uncorrected refractive error	0	0	0	30 (27.5)	2 (18.2)	32 (26.7)
Amblyopia	0	0	0	1 (0.9)	0	1 (0.8)
Total	38 (100)	9 (100)	47 (100)	109 (100)	11 (100)	120 (100)

Visual impairment: low vision plus blindness; Other retinal disease: retinal disease except diabetic retinopathy and ARM; ARM: age-related maculopathy.

Cataracts were also the primary cause of PVA-defined bilateral visual impairment (59.2%), with low vision in 66 of 109 (60.6%) and blindness in 5 of 11 (45.5%) patients. Uncorrected refractive errors were the second most common cause and were found in 32 of 120 (26.7%) individuals with visual impairment. Diabetic retinopathy was the fifth most common cause of bilateral visual impairment, accounting for 2.5% (3/120) of cases based on PVA (Table 4).

Among individuals with unilateral visual impairment, cataracts remained the primary cause, accounting for 56.7% (59/104) of cases based on BCVA and 37.8% (62/164) based on PVA. The second most common causes were corneal opacity (8.7%) based on BCVA and uncorrected refractive errors (31.1%) based on PVA, respectively. Diabetic retinopathy was the eighth most common cause of unilateral visual impairment based on BCVA and PVA (Table 5).

**Table 5:** Causes of unilateral low vision and blindness based on best-corrected visual acuity (BCVA) or presenting visual acuity (PVA) in type 2 diabetes patients in the Dongguan Eye Study (per eye).

Causes	Unilateral low vision and blindness based on BCVA			Unilateral low vision and blindness based on PVA		
	Unilateral low vision (BCVA < 20/63 and ≥20/400) No. at risk (%)	Unilateral blindness (BCVA < 20/400) No. at risk (%)	Unilateral visual impairment (BCVA < 20/63) No. at risk (%)	Unilateral low vision(PVA < 20/63 and ≥ 20/400)No. at risk (%)	Unilateral blindness (PVA < 20/400) No. at risk (%)	Unilateral visual impairment (PVA < 20/63) No. at risk (%)
Cataract	37 (56.9)	22 (56.4)	59 (56.7)	37 (31.4)	25 (54.4)	62 (37.8)
Corneal opacity	2 (3.1)	7 (18.0)	9 (8.7)	2 (1.7)	7 (15.2)	9 (5.5)
Other retinal diseases	7 (10.8)	1 (2.6)	8 (7.7)	7 (5.9)	2 (4.4)	9 (5.5)
Pterygium	6 (9.2)	1 (2.6)	7 (6.7)	8 (6.8)	1 (2.2)	9 (5.5)
Amblyopia	6 (9.2)	0	6 (5.8)	6 (5.1)	0	6 (3.7)
ARM	1 (1.5)	3 (7.7)	4 (3.9)	2 (1.7)	3 (6.5)	5 (3.1)
Uveitis	2 (3.1)	1 (2.6)	3 (2.9)	1 (0.9)	1 (2.2)	2 (1.2)
Diabetic retinopathy	2 (3.1)	0	2 (1.9)	3 (2.5)	1 (2.2)	4 (2.4)
Myopic maculopathy	2 (3.1)	0	2 (1.9)	2 (1.7)	0	2 (1.2)
Optic nerve disease	0	2 (5.1)	2 (1.9)	0	2 (4.4)	2 (1.2)
Glaucoma	0	1 (2.6)	1 (1.0)	0	2 (4.4)	2 (1.2)
Atrophy of eyeball	0	1 (2.6)	1 (1.0)	0	1 (2.2)	1 (0.6)
Uncorrected refractive error	0	0	0	50 (42.4)	1 (2.2)	51 (31.1)
Total	65 (100)	39 (100)	104 (100)	118 (100)	46 (100)	164 (100)

Visual impairment: low vision plus blindness; Other retinal disease: retinal disease except diabetic retinopathy and ARM; ARM: age-related maculopathy.

### Risk factors of visual impairment

In view of the low prevalence of bilateral blindness (0.7% based on BCVA and 0.8% based on PVA), low vision considered together with blindness as visual impairment was assessed via univariate and logistic regression analysis (Tables 6 and 7). As shown in Table 7, visual impairment was positively associated with age, education level and duration of diabetes but was negatively related to BMI based on both BCVA or PVA.

**Table 6:** Univariate analysis of bilateral visual impairment based on best-corrected visual acuity (BCVA) or presenting visual acuity (PVA) in type 2 diabetes patients in the Dongguan Eye Study.

Variables	Based on BCVA			Based on PVA		
	Non-visual impairment (BCVA ≥20/63, n = 1301) No. of persons (%) or Mean ± SD	Bilateral visual impairment (BCVA < 20/63, n = 47) No. of persons (%) or Mean ± SD	*P*	Non-visual impairment (PVA ≥20/63, n = 1228) No. of persons (%) or Mean ± SD	Bilateral visual impairment (PVA < 20/63, n = 120) No. of persons (%) or Mean ± SD	*P*
Gender			0.118			0.001
Male	765 (95.9)	33 (4.1)		518 (94.2)	32 (5.8)	
Female	536 (97.5)	14 (2.5)		710 (89.0)	88 (11.0)	
Age (yrs)						
Mean ± SD	58.9 ± 10.5	77.0 ± 8.6	<0.001	58.3 ± 10.2	71.2 ± 10.8	<0.001
40–49	296 (100.0)	0 (0)	<0.001	291 (98.3)	5 (1.7)	<0.001
50–59	388 (99.5)	2 (0.5)		379 (97.2)	11 (2.8)	
60–69	413 (98.6)	6 (1.4)		395 (94.3)	24 (5.7)	
70–79	157 (88.7)	20 (11.3)		125 (70.6)	52 (29.4)	
≥80	47 (71.2)	19 (28.8)		38 (57.6)	28 (42.4)	
Education			<0.001			<0.001
High school and above	150 (99.3)	1 (0.7)		145 (96.0)	6 (4.0)	
Junior middle school	419 (99.3)	3 (0.7)		412 (97.6)	10 (2.4)	
Primary school	636 (96.8)	21 (3.2)		594 (90.4)	63 (9.6)	
No education	96 (81.4)	22 (18.6)		77 (65.3)	41 (34.7)	
Height (cm)	156.2 ± 8.0	149.4 ± 8.2	<0.001	156.4 ± 8.0	151.1 ± 7.5	<0.001
Weight (kg)	64.4 ± 11.4	52.1 ± 9.3	<0.001	64.8 ± 11.3	55.4 ± 10.1	<0.001
BMI (kg/m^2^)	26.3 ± 3.8	23.4 ± 3.9	<0.001	26.4 ± 3.8	24.3 ± 4.1	<0.001
BSA (m^2^)	1.75 ± 0.18	1.55 ± 0.15	<0.001	1.75 ± 0.18	1.60 ± 0.16	<0.001
Waist circumference (cm)	87.0 ± 9.4	85.0 ± 9.5	0.165	87.1 ± 9.3	84.7 ± 9.5	0.007
Hip circumference (cm)	96.1 ± 7.4	91.9 ± 6.7	<0.001	96.3 ± 7.4	93.4 ± 7.4	<0.001
Waist-hip-ratio	0.90 ± 0.07	0.93 ± 0.08	0.113	0.91 ± 0.07	0.91 ± 0.07	0.770
Heart rate (bpm)	82.5 ± 12.5	88.0 ± 14.1	0.014	82.7 ± 12.4	83.3 ± 14.0	0.574
Mean SBP (mmHg)	141.3 ± 20.0	151.1 ± 21.3	0.001	141.2 ± 20.0	145.4 ± 20.6	0.032
Mean DBP (mmHg)	78.5 ± 11.1	76.2 ± 8.0	0.070	78.8 ± 11.0	74.4 ± 9.7	<0.001
FBG (mmol/l)	7.54 ± 2.87	7.75 ± 2.68	0.635	7.58 ± 2.89	7.22 ± 2.63	0.196
HbA1c (%)	7.05 ± 1.69	6.98 ± 1.42	0.766	7.07 ± 1.71	6.79 ± 1.36	0.041
OGTT (mmol/l)	12.6 ± 6.2	11.9 ± 3.9	0.419	12.6 ± 6.2	11.2 ± 4.3	0.017
TC (mmol/l)	5.46 ± 1.26	5.35 ± 1.32	0.571	5.47 ± 1.25	5.33 ± 1.40	0.300
TG (mmol/l)	1.55 (1.10–2.37)	1.61 (1.13–2.42)	0.881	1.56 (1.11–2.39)	1.44 (1.05–2.26)	0.075
LDL-C (mmol/l)	3.20 ± 1.10	2.94 ± 0.97	0.110	3.21 ± 1.09	3.01 ± 1.14	0.063
HDL-C (mmol/l)	1.40 ± 0.36	1.49 ± 0.41	0.093	1.40 ± 0.36	1.46 ± 0.35	0.065
Scr (μmol/l)	73.3 (61.3–86.1)	74.7 (63.9–94.4)	0.180	73.6 (61.3–86.1)	72.0 (63.3–90.2)	0.488
BUN (mmol/l)	5.78 ± 1.64	6.53 ± 1.86	0.012	5.76 ± 1.63	6.31 ± 1.75	0.002
UA (μmol/l)	377.5 (317.0–453.8)	371.5 (307.3–438.3)	0.612	378.0 (317.5–455.0)	373.0 (308.0–436.0)	0.324

SD: Standard deviation; BMI: body mass index; BSA: body surface area; SBP: systolic blood pressure; DBP: diastolic blood pressure; FBG: fasting blood glucose; HbA1c: hemoglobin A1c; TC: serum total cholesterol; TG: triglycerides; LDL-C: low-density lipoprotein-cholesterol; HDL-C: high-density lipoprotein-cholesterol; Scr: serum creatinine; BUN: blood urea nitrogen; UA: uric acid. BMI = weight (kg)/height (m^2^). BSA (m^2^) = 0.0061 × height (cm) + 0.0124 × weight (kg) − 0.0099. Waist-hip-ratio = waist circumference (cm)/hip circumference (cm).

**Table 7:** Logistic regression analysis of bilateral visual impairment based on best-corrected visual acuity (BCVA) and presenting visual acuity (PVA) in type 2 diabetes patients in the Dongguan Eye Study.

Variable	Bilateral visual impairment based on BCVA (<20/63)		Bilateral visual impairment based on PVA (<20/63)	
	OR (95% CI)	*P*	OR (95% CI)	*P*
Sex (Male vs. Female)	1.27 (0.54–2.99)	0.591	0.84 (0.48–1.45)	0.521
Age (per 10 year)	3.73 (2.39–5.83)	<0.001	2.67 (2.03–3.51)	<0.001
Education*	3.21 (1.63–6.29)	0.001	2.00 (1.35–2.97)	0.001
Duration of diabetes (per year)	1.14 (1.04–1.25)	0.006	1.09 (1.02–1.16)	0.011
BMI (per 1 kg/m^2^)	0.86 (0.77–0.95)	0.005	0.92 (0.86–0.98)	0.009
Hb1AC (per 1%)	1.01 (0.79–1.30)	0.928	0.91 (0.78–1.08)	0.286
SBP (per 10 mmHg)	1.05 (0.85–1.30)	0.657	0.98 (0.86–1.13)	0.801
DBP (per 10 mmHg)	1.23 (0.80–1.88)	0.347	1.01 (0.77–1.32)	0.951
BUN (per 1 mmol/L)	0.90 (0.72–1.12)	0.325	0.96 (0.84–1.10)	0.537

^*^Education was classified as high school or above, junior middle school, primary school and illiteracy; and was assessed as a continuous variable in the regression analysis. Visual impairment: low vision plus blindness. OR: odds ratio. CI: confidence interval. BMI = weight (kg)/height (m^2^).

## Discussion

The current study reports population-based data on the prevalence and causes of low vision and blindness in rural residents of southern China with type 2 diabetes. The prevalence of type 2 diabetes in this population was 16.8%, and the age-standardized prevalences of low vision and blindness were 2.9% and 0.7%, respectively, based on BCVA assessed using World Health Organization (WHO) criteria. The sixth national census in 2010 showed that there were 568.01 million individuals aged 40 years or older in China^10^. Based on our results, we estimate that in the population aged 40 years or older, type 2 diabetes was present in 95.42 million individuals, including 2.77 million patients with low vision and 0.67 million patients with blindness. Based on a rural population of 295.65 million individuals aged 40 years or older^10^, there are an estimated 49.67 million adults with type 2 diabetes living in the rural areas of China, including 1.44 million patients with low vision and 0.35 million patients with blindness.

Although studies on the prevalence of low vision and blindness in general or diabetic populations have been conducted in many countries and regions, their findings cannot be directly compared due to differences in age-selection criteria, race/ethnicity, and the definitions of low vision and blindness. Here, using the same criteria, including a Han Chinese population, rural residence, participants 40 years of age or older, and WHO definitions for visual impairment, we found a slight higher prevalence of low vision and blindness in this population with type 2 diabetes than that in the Beijing Eye Study (1.4% with low vision and 0.5% with blindness based on BCVA)^11^ and the Handan Eye Study (5.9% with low vision and 0.7% with blindness based on PVA)^12^, which focused on the general population in China. These data show indirectly that visual impairment is more likely to occur in diabetic patients than in a general population of adults in mainland China. Among participants aged 60 years or older with diabetes, the prevalence of bilateral visual impairment shown in our study (6.8% based on BCVA and 15.7% based on PVA) is similar to that in the study conducted in a diabetic population from Hong Kong (9.6% based on BCVA and 15.7% based on PVA)^13^ but is markedly lower than that found among Jordanian diabetics (12% with low vision and 14.6% with blindness based on pinhole and glasses-corrected visual acuity)^14^.

Cataracts were the most common cause of low vision and blindness in our population with type 2 diabetes, which is fairly consistent with the findings of studies conducted in a general rural population from mainland China^12, 15, 16^. Cataract surgery is a safe and effective therapy, resulting in satisfactory visual function and low complication rates. Active treatment of a cataract not only improves the patient’s quality of life but also allows for fundus examination, which is significant in the early diagnosis and treatment of diabetic retinopathy and other fundus-related diseases to avoid untreatable blindness as much as possible. However, our questionnaire results indicate that most participants did not know about cataracts and even had a negative attitude towards the treatment. Therefore, it is very important and necessary to strengthen publicity and education about cataracts and increase the rate of cataract surgeries to eliminate blindness caused by cataracts.

The North London Eye Study showed that refractive errors were the second leading cause of presenting visual impairment^17^. Similarly, uncorrected refractive errors were the second most frequent cause of bilateral or unilateral presenting visual impairment in the current study, which indicates that more than a quarter of the type 2 diabetes patients would benefit from an optical prescription. A study conducted in a diabetic population in Hong Kong reported that nearly 70% of cases of visual impairment could be remedied with spectacle correction^13^. There is no doubt that the safest, most economical and easiest approach to improve visual acuity (VA) in patients with refractive errors is to recommend that they wear eyeglasses. Therefore, eye-care providers should educate patients so that they understand the value of good VA, even during the later stages of life when physical activity levels may be reduced.

Diabetic retinopathy is one of the most common complications of diabetes and the leading cause of visual impairment among the working-age populations in North America, Europe, and the Middle East^8, 18–21^. In our study, 950 (70.5%) subjects were newly diagnosed with diabetes and did not have a serious condition or a long disease course, which may be one of the reasons that diabetic retinopathy was not the biggest cause of visual impairment in this population. However, given that diabetic retinopathy is closely related to the duration of diabetes and blood glucose control^21^, it is necessary to focus on active screening, early diagnosis, and timely intervention for diabetic retinopathy and the use of systemic management of diabetes to reduce the risk of untreatable visual impairment. It is worth mentioning that our results show that other retinal diseases (excluding diabetic retinopathy and ARM) were the third leading cause of low vision and blindness. Therefore, greater consideration should be given to these retinal diseases, including retinal vein occlusion, epiretinal membrane, and retinal detachment.

Unilateral low vision and blindness lead to the loss of binocular single vision, along with all of its advantages, including stereopsis, field overlap, exteroception of form and color, and enhanced performance in visuomotor tasks^22, 23^. A person with unilateral visual impairment is at risk of developing bilateral visual impairment and therefore needs special care to prevent or treat visual disabilities in the fellow eye^24^. In this diabetic population, the age-standardized prevalence rate increased from 3.6% for bilateral visual impairment to 7.5% for unilateral visual impairment based on BCVA, while presenting visual impairment increased from 9.0% for bilateral to 11.8% for unilateral visual impairment, suggesting that unilateral low vision and blindness should not be ignored by eye-care providers or patients.

The results of a logistic regression analysis suggest that visual impairment is associated not only with older age, a lack of education and a longer duration of diabetes, as expected, but also with a lower BMI. Lower education levels may imply a lower socioeconomic status and poorer awareness of health- and vision-related diseases. A longer duration of diabetes may indicate more sever diabetic eye complications. These factors are important for vision loss. Given that a higher BMI is generally considered a risk factor for diabetes^25, 26^, additional longitudinal studies are needed to confirm the relationship between BMI and visual impairment.

Some limitations should be considered when evaluating our findings. First, it is difficult to evaluate the main cause of low vision and blindness in some patients objectively and accurately when two or more eye diseases coexist in the same eye; this may introduce a bias in the effect estimates of the causes of low vision and blindness. Second, more than half of the cases of vision impairment were caused by cataracts, corneal opacity, and severe pterygium. Opaque refractive media prevent the clear detection of the fundus, which may cause a possible underestimation of some fundus diseases (e.g., diabetic retinopathy, myopic maculopathy, ARM) and glaucoma. Third, only VA, not the extent of the visual field, was used to evaluate blindness in this study, which may underestimate the prevalence of blindness according to WHO criteria.

In conclusion, this population-based cross-sectional study reports the prevalence and causes of low vision and blindness in a Chinese population with type 2 diabetes aged 40 years or older. The age-standardized prevalence rates of bilateral low vision and blindness were 2.9% and 0.7% based on BCVA and 8.2% and 0.8% based on PVA, respectively. Cataracts were the leading cause of visual impairment. Retinal diseases other than diabetic retinopathy and ARM were the second most common causes based on BCVA, while refraction errors were the second leading cause based on PVA. Our findings indicate that approximately 70% of visual impairment cases in this diabetic population could be eliminated with appropriate surgical therapy or optical correction, such as cataract surgery, pterygium resection, or prescription spectacles. Therefore, eye care providers should not only focus on the diagnosis and treatment of diabetic retinopathy but should also give greater consideration to patient education and the screening of common eye diseases to reduce the risk of low vision and blindness in the rural southern Chinese population, especially in older diabetic patients with lower levels of education.

## Methods

### Study design, participants and procedures

The DES was a population-based study of the frequency and risk factors of visual impairment and the major vision-threatening eye diseases in an adult rural population, aged 40 years or older, from Hengli Town of Dongguan City in southern China, which was conducted from September 2011 to February 2012^27^. This study complied with the Declaration of Helsinki, and was approved by the Medical Ethics Committee of Dongguan People’s Hospital and Research Ethics Committee of Guangdong General Hospital. Each step in the study was verbally explained to the participants, and a written description of the study and a consent form were provided to the participant. For participants who could not read due to vision loss or illiteracy, the consent statement was read to them by the interviewer. Informed consent with a signature or handprint was obtained from all study subjects for sampling and research. All the methods were carried out in accordance with relevant approved guidelines and regulations.

Residents aged 40 years or older living in the one community and 16 administrative villages in Hengli Town of Dongguan were enrolled in the DES. Based on the inclusion criteria^27^, 11,357 individuals were confirmed as eligible for inclusion. A total of 8952 (78.8%) residents were recruited to take part in systemic and ophthalmic examinations, and 1500 of them with type 2 diabetes according to the American Diabetes Association diagnostic criteria (2010)^28^ were screened. Of these diabetic patients, 1348 (89.9%) completed the eye examinations, and their data were analyzed in this study. The detailed design, survey methods, procedures, methods of examination, and baseline characteristics of participants in the DES have been previously reported^27^. Briefly, demographic, socioeconomic status, and health- and vision-related quality of life data were collected during interviews. Physical measurements included height, weight, waist and hip circumference, heart rate, and blood pressure. Laboratory tests included fasting blood glucose, hemoglobin A1c, oral glucose tolerance, serum total cholesterol, triglyceride, high-density lipoprotein-cholesterol, low-density lipoprotein-cholesterol, serum creatinine, blood urea nitrogen, and uric acid measurements. Ophthalmic examinations included a basic and specialized inspection.

### Ophthalmic examinations

The basic ophthalmic examinations included ocular history, VA and autorefraction testing (ARK-700A, NIDEK, Hiroishi, Japan), intraocular pressure measurements (AT80, Topcon, Tokyo, Japan), and anterior and posterior segment examinations (slit-lamp biomicroscope, IEC 601-1, SL-1E, Topcon, Japan; 90-diopter lens, Volk, Mentor, OH, USA). Distance VA was measured monocularly using an International Standard Visual Acuity Chart with a standard illumination box at a distance of 5 m. The identifying time for each optotype was 3 seconds^29^. If no optotypes were read at 5 m, the participant was asked to move closer to the chart. VA was quantified according to the following formula: VA = 0.1 × distance/5^29^ and recorded as 0.02 at 1 m. If no optotypes were identified on the chart, VA was assessed based on counting fingers, hand movements, and light perception or no light perception along with the examination distance. BCVA was determined using autorefraction results and PVA was determined based on habitual refractive correction. VA was recorded in decimal units and was converted to the Snellen fraction or the logarithm of the minimal angle of resolution (logMAR) if necessary.

Specialized ophthalmic examinations were conducted in the following subject groups. For subjects with a BCVA less than 20/32 (decimal VA, 0.6) that could not be explained by corneal and lens diseases or in cases with suspected fundus diseases and optic neuropathy, the pupil was dilated using tropicamide compound eye drops (containing 5 mg of tropicamide and 5 mg of phenylephrine hydrochloride per milliliter), followed by fundus examination and photography. In patients with shallow anterior chambers and angle-closure glaucoma who had not undergone laser or surgical treatment, pupillary dilation was avoided. For patients diagnosed with diabetes mellitus and hypertension, non-mydriatic fundus photography was performed, and some patients with refractive media opacity underwent mydriatic fundus photography. In patients with severe non-proliferative or proliferative diabetic retinopathy and those suspected of having macular edema, retinal vascular lesions, posterior uveitis or ARM, fundus fluorescein angiography was performed. In cases with an undetectable posterior segment during routine fundus examination or with a suspected intraocular tumor, B-mode ultrasonography (ODM-2100S Ultrasonic A/B Scanner, MEDA, Tianjin, China) was performed. Patients with suspected glaucoma (limbal anterior chamber depth ≤25% of limbal corneal thickness under a slit-lamp biomicroscope, vertical cup-to-disc ratio [VCDR] ≥0.7 in either eye, VCDR asymmetry ≥0.2, intraocular pressure ≥21 mmHg, or a family history of glaucoma) underwent gonioscopy, central corneal thickness measurement, fundus photography, visual field testing, and retinal nerve fiber layer imaging.

### Definition of low vision and blindness

Low vision and blindness were defined based on WHO criteria^30^. Low vision was present if VA was worse than 20/63 (decimal VA, 0.3) but better than or equal to 20/400 (decimal VA, 0.05) in the better eye, and blindness was defined as VA worse than 20/400 (decimal VA, 0.05) in the better eye. In addition to reporting low vision and blindness in terms of the better eye (bilateral low vision and blindness), data on unilateral low vision (defined as VA of less than 20/63 but equal to or better than 20/400) and unilateral blindness (defined as VA worse than 20/400) in the worse-seeing eye are also presented. In the present study, low vision and blindness were assessed based on BCVA and PVA. Low vision includes moderate visual impairment and severe visual impairment as defined in the International Classification of Diseases −10 (Update and Revision 2006)^31^, and visual impairment includes low vision and blindness.

### Causes of low vision and blindness

The principal cause of low vision or blindness was determined by two expert ophthalmologists (Y. C. and Q. M.) based on general and ocular records. In cases of disagreement, consensus was reached by discussion with a third ophthalmologist (M. Z.). For eyes with two or more diseases that may have caused low vision or blindness, the cause that had the presumed greatest impact on visual impairment was regarded as the primary diagnosis.

### Statistical analysis

Statistical analysis was performed using SPSS software (version 20.0; SPSS Inc., Chicago, IL, USA). The crude prevalence of low vision and blindness was described using prevalence rates and 95% CIs. Age-standardized prevalence of low vision or blindness and 95% CIs were estimated by standardizing of the study sample to the Chinese population of type 2 diabetes aged 40 years or older, which was calculated based on the Chinese population information from the Sixth National Census^32^ and the prevalence of diabetes in the Chinese adult population^33^. Demographic and physical characteristics are expressed as frequencies for categorical variables and the mean ± standard deviation for continuous variables. Proportions were compared using chi-square tests for non-ordinal data and Wilcoxon rank-sum tests for ordinal data. Continuous variables were tested using *t*-tests or one-way ANOVAs, as appropriate. Multivariate logistic regression analyses were conducted to determine the factors influencing visual impairment in patients with type 2 diabetes.
